# Supplementary material for: Perforin inhibition protects from lethal endothelial damage during fulminant viral hepatitis
Source: Nat Commun. 2018 Nov 15;9:4805. doi: 10.1038/s41467-018-07213-x (PMC6237769; doi:10.1038/s41467-018-07213-x)
Supplement: Supplementary file 1 — Supplementary Information [file 41467_2018_7213_MOESM1_ESM.pdf]

Perforin inhibition protects from lethal endothelial damage during fulminant viral hepatitis

Welz et al.

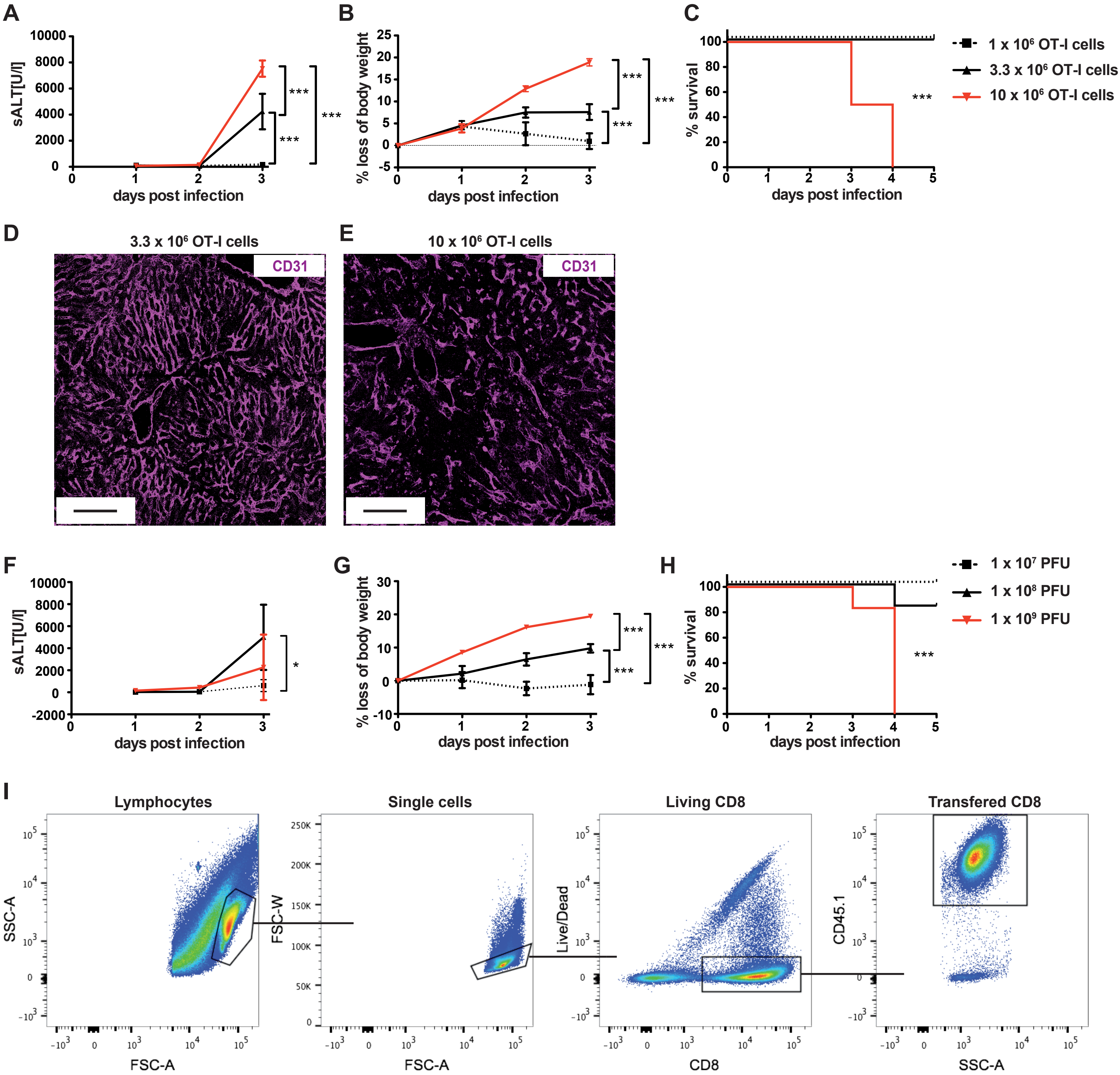

Supplementary Figure 1: Titration of the number of transferred OT-I cells and virus
(A/F) shows ALT levels in the serum, (B/G) shows the percent loss of body weight over time and (C/H) shows the survival curve of mice transferred with 1 x 10^6, 3.3 x 10^6 or 10 x 10^6 OT-I cells and infected with 1 x 10^9 PFU AdGOL (A/B/C) or transferred with 7 x 106 OT-I cells and infected with 1 x 10^7, 1 x 10^8 or 1 x 10^9 PFU AdGOL. (I) shows gating strategy to identify transfered CD8 T cells. Data is representative of two independent experiments (n=6). Error bars indicate the mean ± SD. Comparison between groups was calculated using a one-way ANOVA with a Tukey's multiple comparison post test (A/B/F/G) or a log-Rank test (C/H). * = $p \leq 0.05$; *** =$p < 0.001$.

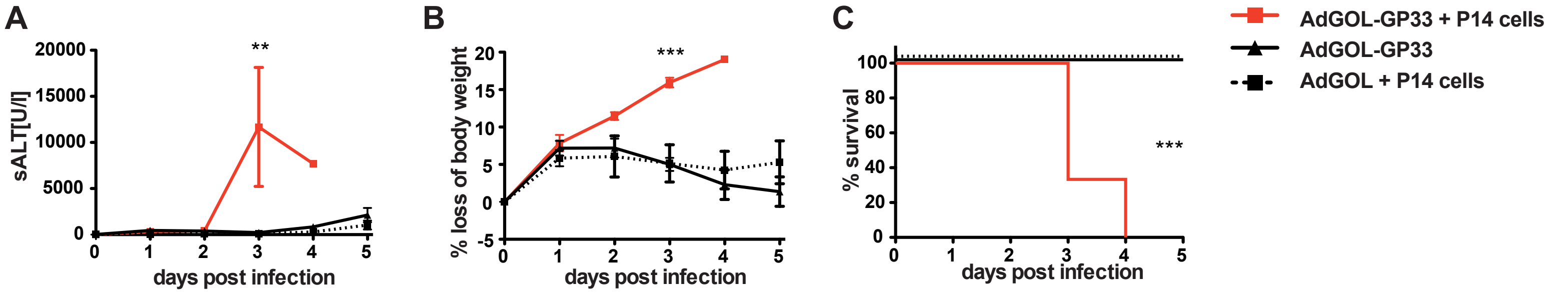

Supplementary Figure 2: Model of CD8⁺ T cell mediated acute liver failure using GP33 as an antigen
(A) shows ALT levels in the serum, (B) shows the percent loss of body weight over time and (C) shows the survival curve of mice transferred with 7 x 10^6 P14 cells and infected with 1 x 10^9 PFU AdGOL-GP33 (n=5).
Error bars indicate the mean ± SD. Comparison between groups was calculated using a one-way ANOVA with a Tukey's multiple comparison post test (A/B) or a log-Rank test (C). ** = $p < 0.01$; *** =$p < 0.001$.

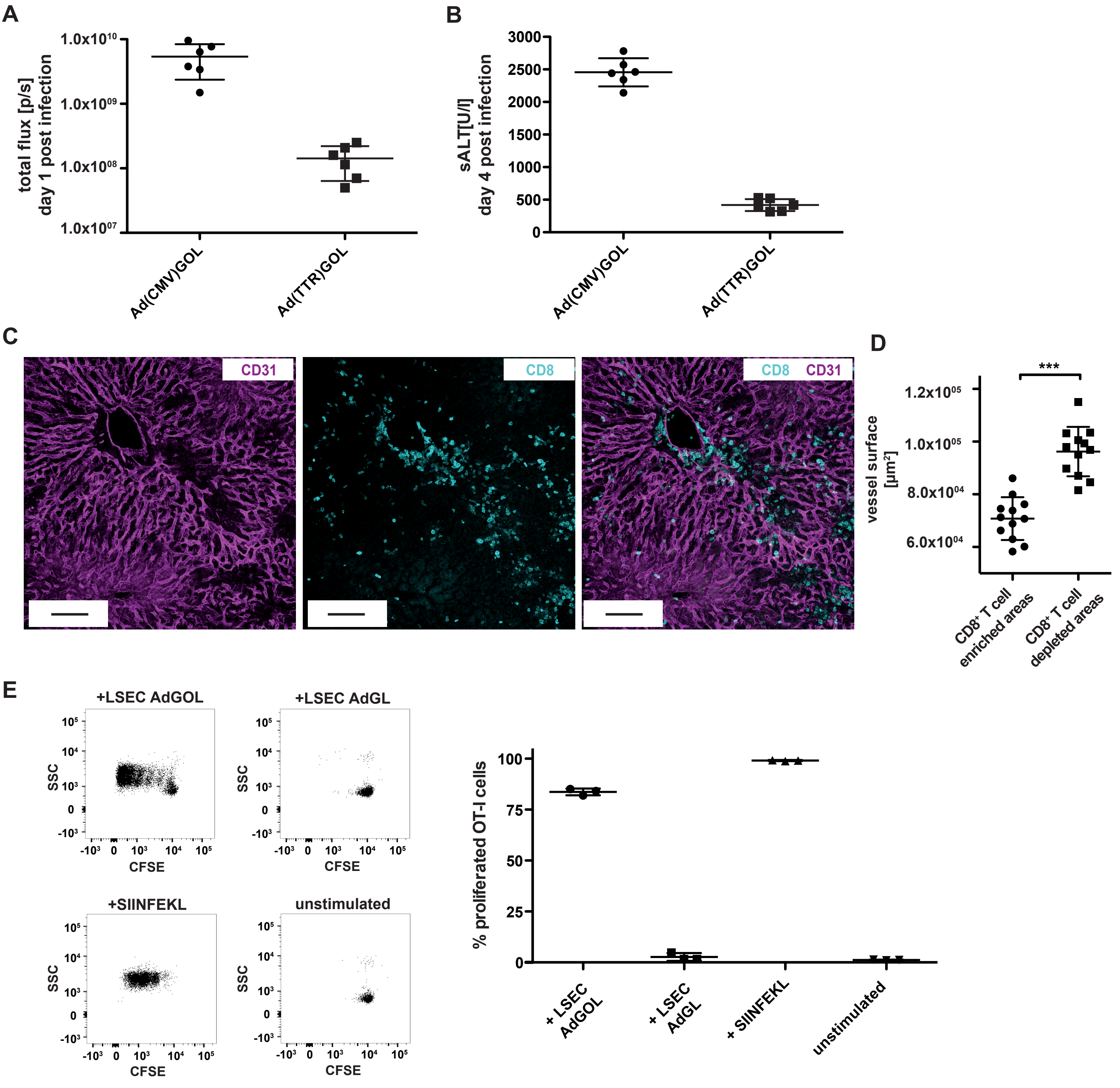

Supplementary Figure 3: LSEC become CTL targets via cross-presentation of viral antigen.
(A/B) *In vivo* luciferase activity (A) and serum ALT levels (B) comparing AdGOL driven under the CMV or TTR (hepatocyte-specific) promoter.
(C) Immunofluorescence images showing CD31 staining and quantification of vessel surfaces (D) of livers on d3 after transfer of CD44$^-$OT I cells and infection with $1x10^9$ PFU Ad(TTR)GOL. (E) CFSE profiles and proliferation rates of OT-I T cells conicubated with LSEC isolated from livers on day 3 after infection with $1x10^9$ PFU AdGOL or $1x10^9$ PFU AdGL. Data is representative of two (E) or three (A-D) independent experiments (n=3).
Scale bars: 80µm, Error bars indicate the mean ± SD. Comparison between groups was calculated using the unpaired student's t-test. *** =p < 0.001.

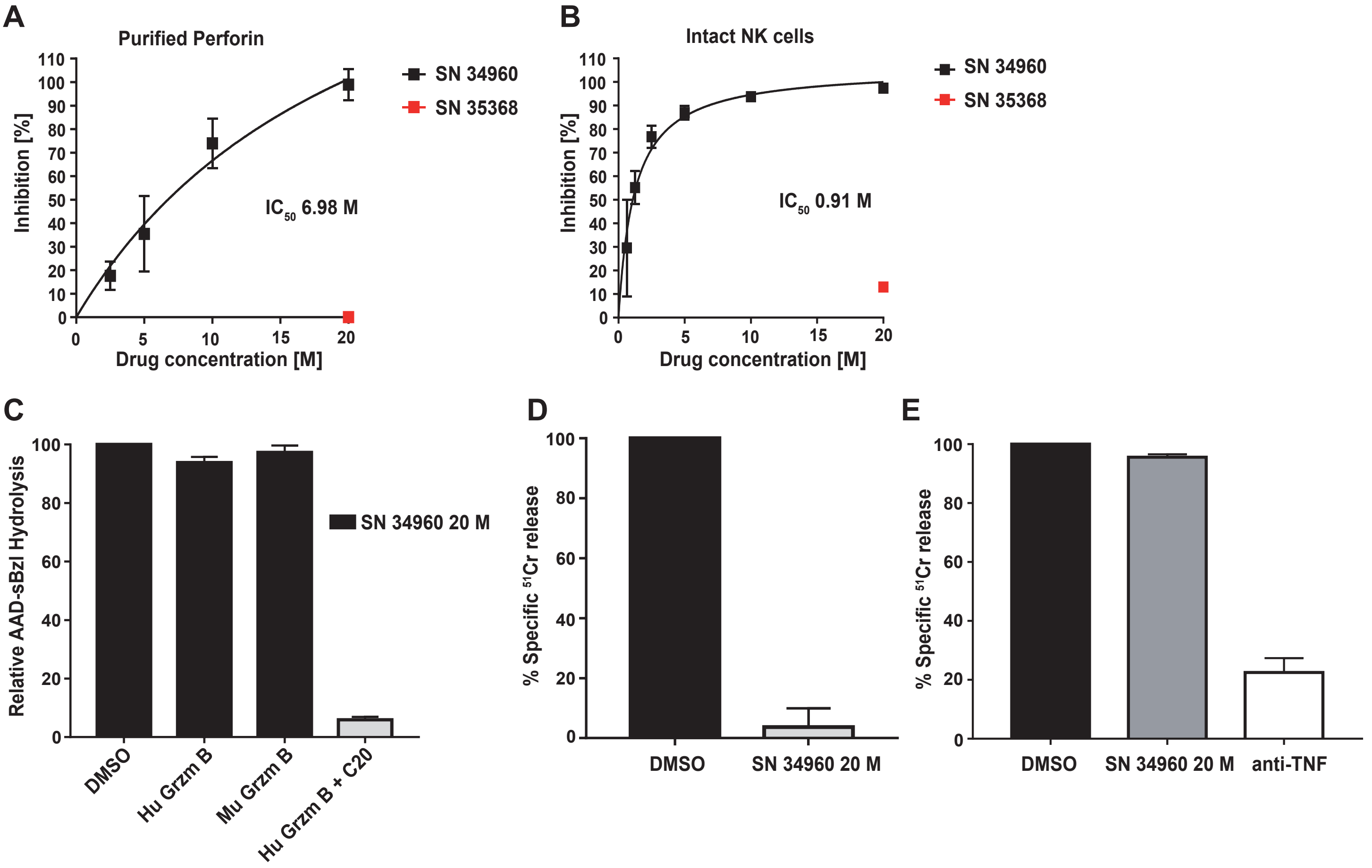

Supplementary Figure 4: ***In vitro*** **analysis of perforin inhibitor compounds.**
(A) Inhibition of lysis by purified recombinant perforin. Chromium release assay (4h) using 51Cr-labelled Jurkat cells and recombinant mouse perforin (5nM). Recombinant perforin was pre-incubated for 30 mins with DMSO or perforin inhibitor SN 34960 or a similar but inactive compound SN 35368 (20µM) followed by incubation with Jurkat cells at 37°C for 4 hrs as described before[1]. The data are pooled from n=3 independent experiments, with each data point in each experiment assayed in triplicate.
(B) Inhibition of cytotoxicity by intact NK cells. Chromium release assay (4h) using KHYG1 NK cells as effectors and 51Cr-labelled K562 cells as targets at an effector to target ratio (E:T) of 16:1. KHYG1 cells were pre-incubated for 30mins with perforin inhibitor SN 34960 or a similar but inactive compound SN 35368 (20µM) followed by incubation with K562 target cells at 37°C for 4 hrs as described in (JA Spicer et al., 2017). The data are pooled from n=3 independent experiments, with each data point in each experiment assayed in triplicate.
(C) Perforin inhibitor SN 34960 does not block Granzyme B proteolytic activity. The proteolytic activity of human and mouse granzyme B was assigned at 100% as the maximum rate of hydrolysis of Boc-Ala-Ala-Asp-S-Bzl in the presence or absence of perforin inhibitor SN 34960 (20µM) or the specific Granzyme B inhibitor C20 (10µM) as described before[2]. The data are pooled from n=3 independent experiments, with each data point in each experiment assayed in triplicate.
(D) Inhibition of perforin dependent, granzyme-mediated apoptosis by SN 34960**.** Recombinant low dose mouse perforin was pre-incubated with perforin inhibitor SN 34960 or DMSO for 15mins followed by incubation with mouse granzyme B (120nM) and target cells at 37°C for 4 hrs. The data are pooled from n=3 independent experiments, with each data point in each experiment assayed in triplicate.
(E) SN 34960 does not inhibit TNF-mediated apoptosis. Recombinant TNF (30ng/ml) was pre-incubated with either SN 34960, DMSO or anti-TNF (10µg/ml) for 30mins followed by addition of 51Cr- labelled MC38 target cells at 37°C for 4 hrs. The data are pooled from n=3 independent experiments, with each data point in each experiment assayed in triplicate.

Supplementary References:

1. Spicer JA, Miller CK, O'Connor PD, Jose J, Huttunen KM, Jaiswal JK, Denny WA, Akhlaghi H, Browne KA, Trapani JA. Benzenesulphonamide inhibitors of the cytolytic protein perforin. *Bioorg Med Chem Lett.* **27**(4),1050-1054 (2017).

2. Willoughby CA, Bull HG, Garcia-Calvo M, Jiang J, Chapman KT, Thornberry NA. Discovery of potent, selective human granzyme B inhibitors that inhibit CTL mediated apoptosis. *Bioorg Med Chem Lett*. **12**(16), 2197-200 (2002).
