# Supplementary material for: Perforin inhibition protects from lethal endothelial damage during fulminant viral hepatitis
Source: Nat Commun. 2018 Nov 15;9:4805. doi: 10.1038/s41467-018-07213-x (PMC6237769; doi:10.1038/s41467-018-07213-x)
Supplement: Supplementary file 3 — Description of Additional Supplementary Files [file 41467_2018_7213_MOESM3_ESM.pdf]

Description of Additional Supplementary Files

File Name: Supplementary Movie 1
Description: Movie shows distribution of Evans blue in the liver *in vivo* after infection 2x109 PFU AdGOL i.v. (d-2) and injection with PBS (-4hrs) i.v. Data is representative of two independent experiments (n=3).

File Name: Supplementary Movie 2
Description: Movie shows distribution of Evans blue in the liver *in vivo* after infection 2x109 PFU AdGOL i.v. (d-2) and injection with 0.4 µg TNF (-4hrs) i.v. Data is representative of two independent experiments (n=3).

File Name: Supplementary Movie 3
Description: Movie shows distribution of Evans blue in the liver *in vivo* 3 days after transfer of 7x106 OT-I T cells and infection with 1x109 PFU AdGL. Data is representative of three independent experiments (n=3).

File Name: Supplementary Movie 4
Description: Movie shows distribution of Evans blue in the liver *in vivo* 3 days after transfer of 7x106 OT-I T cells and infection with 1x109 PFU AdGOL. Data is representative of three independent experiments (n=3).

File Name: Supplementary Movie 5
Description: Movie shows intravital microscopy of in the livers 3 days after transfer of 7x106 OT-I T cells and infection with 1x109 PFU AdTL. Mice were injected with 2µg fluorescent anti-GP1bß antibody to visualize platelets. Data is representative of three independent experiments (n=3).

File Name: Supplementary Movie 6
Description: Movie shows intravital microscopy of in the livers 3 days after transfer of 7x106 OT-I T cells and infection with 1x109 PFU AdTOL. Mice were injected with 2µg fluorescent anti-GP1bß antibody to visualize platelets. Data is representative of three independent experiments (n=3).
